# Feasibility of Nitrogen Doped Ultrananocrystalline Diamond Microelectrodes for Electrophysiological Recording From Neural Tissue

**DOI:** 10.3389/fbioe.2018.00085

**Published:** 2018-06-22

**Authors:** Yan T. Wong, Arman Ahnood, Matias I. Maturana, William Kentler, Kumaravelu Ganesan, David B. Grayden, Hamish Meffin, Steven Prawer, Michael R. Ibbotson, Anthony N. Burkitt

**Affiliations:** ^1^Department of Physiology and Department of Electrical and Computer Systems Engineering, Biomedicine Discovery Institute, Monash University, Melbourne, VIC, Australia; ^2^School of Physics, University of Melbourne, Melbourne, VIC, Australia; ^3^Department of Biomedical Engineering, University of Melbourne, Melbourne, VIC, Australia; ^4^National Vision Research Institute, Australian College of Optometry, Carlton, VIC, Australia; ^5^Department of Optometry and Vision Science University of Melbourne, Melbourne, VIC, Australia

**Keywords:** ultrananocrystalline diamond, N-UNCD, microelectrode array, neural prostheses, electrophysiology

## Abstract

Neural prostheses that can monitor the physiological state of a subject are becoming clinically viable through improvements in the capacity to record from neural tissue. However, a significant limitation of current devices is that it is difficult to fabricate electrode arrays that have both high channel counts and the appropriate electrical properties required for neural recordings. In earlier work, we demonstrated nitrogen doped ultrananocrystalline diamond (N-UNCD) can provide efficacious electrical stimulation of neural tissue, with high charge injection capacity, surface stability and biocompatibility. In this work, we expand on this functionality to show that N-UNCD electrodes can also record from neural tissue owing to its low electrochemical impedance. We show that N-UNCD electrodes are highly flexible in their application, with successful recordings of action potentials from single neurons in an *in vitro* retina preparation, as well as local field potential responses from *in vivo* visual cortex tissue. Key properties of N-UNCD films, combined with scalability of electrode array fabrication with custom sizes for recording or stimulation along with integration through vertical interconnects to silicon based integrated circuits, may in future form the basis for the fabrication of versatile closed-loop neural prostheses that can both record and stimulate.

## Introduction

Over the past decades, growing numbers of prostheses that can interact with the central and peripheral nervous systems have become clinical realities. New varieties of neural prostheses that function through electrical stimulation of the nervous system have emerged, addressing conditions such as epilepsy (Heck et al., 2014), Parkinson's disease (Deuschl et al., 2006), vision or auditory impairment (Grayden and Clark, 2006; Wilson and Dorman, 2008; Zrenner et al., 2011; Ayton et al., 2014; Clark, 2015; Ho et al., 2015), and urinary bladder function (Gaunt and Prochazka, 2006). Similarly, devices capable of restoring touch sensation by stimulating the sensorimotor cortex (O'Doherty et al., 2011) and peripheral nerves of the arm (Dhillon and Horch, 2005) have been demonstrated for upper limb brain-machine interface applications.

Concurrently, significant progress has been made toward neural prostheses that record neural activity to infer a user's intentions to control devices such as robotic arms (Collinger et al., 2012; Hochberg et al., 2012) or a cursor on a screen (Gilja et al., 2012). These developments in neural signal recording and processing have been incorporated in neuronal stimulation prostheses to provide a mechanism for implementing closed-loop feedback for implants. In these systems, the recorded neural signals are used to adjust online and in real-time the most appropriate stimulation for an individual user's current physiological state (Rosin et al., 2011; Berényi et al., 2012; Heck et al., 2014).

Microelectrode arrays have been commonly used to interface with biological tissue to stimulate and to record (Warren et al., 2016; Rosenfeld and Wong, 2017). Many of these devices employ large numbers of electrodes, with sizes comparable to those of the targeted neuronal cells (Viventi et al., 2011). Greater control of prostheses is facilitated using microelectrode arrays with high electrode density and many electrodes, as they have the potential to extract more information from the neural tissue and to provide more spatially selective stimulation as part of a closed loop. However, the increase in the electrochemical impedance of the electrodes with the reduction in their physical size typically results in a higher recording signal-to-noise (SNR) ratio, lower stimulation charge injection capacity, and a more challenging stimulation artifact interference (Merrill et al., 2005; Spira and Hai, 2013). Moreover, current technologies are limited, in part, due to the availability of a suitable device architecture that can provide the high count and high density hermetic feedthroughs required to connect microelectrode arrays to encapsulated electronic circuitry. This problem becomes increasingly difficult for devices with more than 100 electrodes (Weiland and Humayun, 2008; Stieglitz, 2010). Novel devices with new materials are needed to enable high channel count and density neuronal interfacing devices.

In earlier work, we described an all-diamond microelectrode array implant architecture (Ahnood et al., 2017) that is compatible with both high count and high density electrodes, which uses vertical interconnects (Ahnood et al., 2015) and feedthroughs (Ganesan et al., 2014). The array consists of nitrogen doped nanocrystalline diamond (N-UNCD) electrodes embedded in an insulating polycrystalline diamond (PCD) matrix with vertical hermetically sealed feedthroughs (as shown via helium leak tests), and provides a solution to both the electrode number and feedthrough scalability issues (Ganesan et al., 2014). The minimum electrode size (~10 μm) and density with N-UNCD is determined by the microfabrication technology (in this case, the 5 μm resolution of laser micromachining) as well as flip-chip bonding resolution for interconnection with the integrated circuit (Ahnood et al., 2015). In addition, N-UNCD electrode materials have been shown to be biocompatible (Garrett et al., 2016) with surface treatments such as oxygen plasma activation and roughening increasing stability and efficacy of the tissue-electrode interface (Tong et al., 2016). All these features make N-UNCD a potential candidate for use in stimulating and recording neural prostheses.

It has also been shown that N-UNCD can be integrated with a stimulating Application Specific Integrated Circuit (ASIC) to fabricate high electrode count and high electrode density arrays (Ganesan et al., 2014; Ahnood et al., 2015, 2017) to form a hermetically sealed stimulating neural prosthesis (Lichter et al., 2015). Furthermore, the material has been shown to be suitable for neuronal stimulation (Hadjinicolaou et al., 2012), with high stability and charge injection capacity and a wide window before water splitting occurs. Square N-UNCD electrodes of size 200 × 200 μm are reported to have a charge injection capacity of 250 μC/cm^2^, which is higher than that of typical platinum electrodes (~150 μC/cm^2^, Cogan, 2008; Hadjinicolaou et al., 2012). However, no study to date has examined the recording capabilities of this material and evaluated its suitability for recording neural prostheses.

In the present paper, we demonstrate that N-UNCD can successfully record neural signals in both *in vivo* and *in vitro* preparations. As such, we show that N-UNCD is a suitable material for fabrication of a high density, high channel count, closed-loop feedback neural prostheses, with the capability to provide both neuronal stimulation and recording. Specifically, we report on the electrochemical and electrophysiological performance of N-UNCD microelectrodes for measurements from the visual cortex *in vivo* as well as from the retina *in vitro*.

## Methods

### Fabrication of recording and stimulating electrodes

The electrodes were fabricated from N-UNCD within an insulating PCD substrate with silver braze metallic feedthroughs for vertical electrical connection. The fabrication steps for stimulating the N-UNCD electrode array were reported by Ahnood et al. (2015) and adopted here, with the key difference of incorporating additional electrodes for recording. In summary, to fabricate the arrays, PCD wafers (TM100 grade, Element 6 Ltd) were purchased and electrode holes were milled into the surface using an Nd:Yag laser (532 nm, Alpha series, Oxford Lasers). The holes were then filled with conductive N-UNCD to form the electrodes via microwave chemical vapor deposition (Cyrannus 1 Plasma source, Iplas Innovaive Plasma Systems gmbh). Finally, to electrically isolate electrodes between each feedthrough channel, the area between electrodes was laser milled and then plasma treated (50 W 3:1 Ar:O_2_) to remove any conducting materials that may have been formed from the milling. After fabrication, all electrode arrays were inspected using a scanning electron microscope (Nova NanoSEM, FEI) to ensure quality of fabrication.

**Figure 1:**
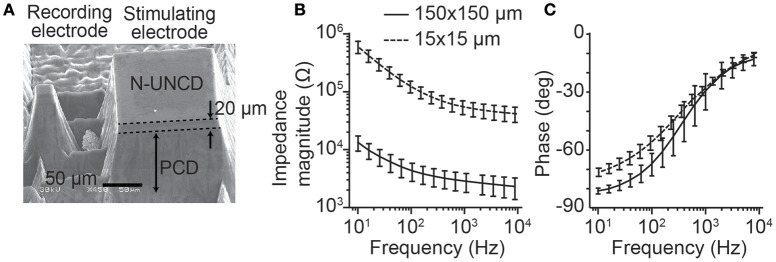
Stimulating and recording microelectrode manufactured from N-UNCD and the electrochemical impedance spectroscopy. **(A)** A pair of stimulating and recording electrodes. The stimulating electrodes have top surface dimensions of 150 × 150 μm, while the recording electrodes were 15 × 15 μm. The total height of the electrodes was 110 μm with only the tip of the electrode (~20 μm) being conductive. **(B)** The magnitude of the impedance (mean ± std) for the smaller recording electrodes was greater than that of the stimulating electrodes. **(C)** The phases of the impedances for the same electrodes showed a smaller difference (mean ± std).

To record from neural tissue, we manufactured a single recording and stimulating electrode on a carrier that could be easily placed on the cortical or retinal surface (Figure 1A). Electrodes protruded from the surface of the PCD to allow for improved contact with the neural tissue. The stimulating electrodes had dimensions 150 × 150 × 110 μm (width × height × depth), while the recording electrodes were smaller with dimensions 15 × 15 × 110 μm. For both electrode types, only the tip of the shank was fabricated to be electrically conductive (top end with a height of 20 μm) to maximize the ability to record or stimulate locally. To allow for flexibility in the choice of neural recording systems used in the *in vivo* and *in vitro* experiments, wire bonding was used to connect each electrode to a connector.

To demonstrate the scalability of the technology in terms of electrode count and density an array of 16 × 16 stimulating electrodes was also manufactured (section Characterization of Focused Electrical Stimulation). These electrodes were characterized on the benchtop in saline and were not used to record or stimulate from neural tissue. Electrode sizes match those described in the section above.

### Characterization of the electrode recording capabilities

All animal experiments conformed to the policies of the National Health and Medical Research Council of Australia and were approved by the Animal Experimentation Ethics Committee of the University of Melbourne, Faculty of Science (Approval Number: 1112084 and 1413312).

### Cortical recordings

The neural recording capabilities of a 15 μm N-UNCD recording electrode were characterized via *in vivo* visual stimulation experiments in felines (*n* = 2). The animals were initially anesthetized with a single injection of ketamine (20 mg/kg, i.m.) and xylazine (1 mg/kg, i.m.) and then intubated (van Kleef et al., 2010). Anesthesia was maintained with gaseous halothane (0.5–1%) and the animal was mechanically ventilated. Pupils were dilated with atropine (1%) and phenylephrine (10%), and gallamine triethiodide (10 mg/kg/hr, i.v.) was delivered to reduce eye movement. Finally, a craniotomy was performed over visual cortex (areas 17 and 18) in the hemisphere contralateral to the stimulated eye (Tusa et al., 1978) and a durotomy performed. A single recording electrode was lowered onto the surface of the cortex with the aid of a stereotaxic mounted manipulator (David Kopf Instruments, USA). Data was filtered between 0.01 Hz−10 kHz and sampled at 30 kHz (Spike2, Cambridge Electronic Design, UK). Recordings were referenced to a platinum return electrode placed in the temporal muscle.

Visual stimuli were delivered via a gamma-corrected monitor (ASUS VG248) with stimuli consisting of large field, square wave drifting gratings presented in one of eight equally spaced orientations (spatial frequency, 0.2 cycles/degree, Michelson contrast, *c* = 1, temporal frequency, 2 cycles/s). Twenty trials of each direction were repeated in a randomized order. Gratings were produced by a ViSaGe visual stimulus generator (Cambridge Research Systems Ltd., UK) and each was initially presented and kept stationary for 0.5 ms and then moved for 2 s. Stimuli were presented with a 3 s delay between each grating with an isoluminant gray screen at a level matched to the mean luminance of the gratings presented during these times. Control trials in which the screen was left blank were interleaved randomly between stimulus trials. The signal-to-noise ratio (SNR) of the recordings was calculated as the ratio between the root mean square values of the signal amplitude in a 500 ms window directly after the stimulus presentation relative to a 500 ms window directly preceding the presentation.

### Retinal recordings

The recording capabilities of the N-UNCD electrodes were also tested *in vitro* with retinal whole mount preparations. Data were acquired from retinae of Long-Evans rats ranging from 1 to 6 months of age (*n* = 3). The animals were initially anesthetized with a mixture of ketamine and xylazine prior to enucleation. After enucleation, the rats were euthanized with an overdose of pentobarbital sodium (350 mg intracardiac). Dissections were carried out in dim light conditions to avoid bleaching the photoreceptors. After hemisecting the eyes behind the ora serrata, the vitreous body was removed, and each retina was cut into four pieces. The retinae were left in a perfusion dish with carbogenated Ames medium (Sigma) at room temperature until used. Pieces of retina were mounted on a glass coverslip with ganglion cell layer up and were held in place with a perfusion chamber and stainless steel harp fitted with Lycra threads (Warner Instruments). Once mounted in the chamber, the retina was perfused (4–6 mL/min) with carbogenated Ames medium (Sigma-Aldrich, St. Louis, MO) at 32–34°C. The chamber was mounted on the stage of an upright microscope (Olympus Fluoview FV1200).

Extracellular recordings were obtained with a 15 μm N-UNCD electrode. Signals were acquired and amplified (Tucker Davis Technologies: RZ2 base station and PZ2 multichannel recorder), filtered online with band-pass frequencies between 5 and 15 kHz, and digitized at 50 kHz for offline analysis. Light responses were obtained by using a full field light to illuminate the retina. The mean number of spikes recorded during light-on and light-off periods were analyzed. Additionally, the instantaneous changes in responses from 1 s prior compared to 1 s after a change in illumination were also analyzed. Putative spikes were detected when the recorded signal crossed a threshold set at 3.5 standard deviations above the baseline noise. A spike cluster analysis (Quiroga et al., 2004) was used to classify and group spike waveforms with similar shapes. Once clustered, the SNR was calculated to evaluate the quality of the recordings. The SNR was calculated by taking the ratio of the amplitude of the average spike waveform to the standard deviation of the spike waveform noise (Kelly et al., 2007).

### Characterization of focused electrical stimulation

To demonstrate the ability of N-UNCD to form a high channel count and high density neural prosthesis, a 256 (16 × 16) electrode stimulating array was fabricated. This array was then bonded to an Application Specific Integrated Circuit (ASIC) capable of stimulating through each electrode, with the addition of three power smoothing and conditioning capacitors (**Figure 5**). The ASIC was controlled and powered externally through a three-wire interface. These wires were used for forward and backward telemetry and power delivery from an external driving circuit, and were terminated through an adaptor onto the diamond device by wire-bonding. The details and electrical functional testing of the ASIC are described by Tran et al. (2014).

To connect the 256-channel electrode array to a stimulating ASIC, the electrode array was flip-chip bonded and then potted in epoxy resin (Ahnood et al., 2015). Wire bonding was used to connect the assembled device to a computer interface unit (**Figure 5**). External power conditioning capacitors were directly soldered onto the diamond array contact pads. To test for correct bonding of the N-UNCD electrodes to the ASIC, the device was submerged in saline and the ASIC was used to drive charge through each electrode sequentially against a distant return electrode. Using on-board circuitry on the ASIC, voltages across the electrodes were measured to ensure that there was an electrical path from the ASIC through the N-UNCD electrodes for each current source. To ensure that each electrode was not shorted to other electrodes through the internal circuitry, the device was placed in air and current was sequentially delivered through neighboring electrodes and the voltage measured on board to ensure that no current flowed through the device.

To test the functionality of the high-density array and ASIC stimulator, voltage changes were monitored in a saline bath while charge was delivered through the electrodes. The electrode and ASIC were mounted onto a printed circuit board (PCB) carrier and a cylindrical chamber with an inner diameter of 15 mm was mounted on top of the PCB. The chamber was filled with 1 ml of 0.9% saline solution. Biphasic stimuli with amplitudes of 60 or 20 μA and equal phase durations of 500 μs and an interphase gap of 250 μs were passed through selected electrodes. Stimulation delivered via the ASIC was controlled through a custom three-wire communication protocol provided by a microcontroller (LPC1759, Semiconductors) that, in turn, was driven by a PC running custom MATLAB code.

To record the voltage changes in the saline bath, a polyimide-coated tungsten microelectrode (Alpha Omega) with a minimum impedance of 1 MΩ was used to scan voltage data at different locations relative to a platinum return electrode placed 5 mm from the edge of the array. The tungsten microelectrode was moved in fixed 200 μm step increments above the surface of the electrode array using a three-axis micromanipulator (MPC-200 and ROE-200, Sutter Instrument Company Ltd) controlled through custom MATLAB software (Multi-Link, Sutter Instrument Company Ltd). The electrode was positioned ~100 μm from the surface of the electrode array. The voltage data was band-pass filtered between 1 Hz and 10 kHz and sampled at 30 kHz (Cambridge Electronic Design Ltd). A minimum of 10 biphasic pulses were sampled at each location and the average peak-to-trough voltage calculated.

## Results

We present multiple results to highlight the functionality of the N-UNCD electrodes. First, we present bench-top impedance characterizations of the device, and then show its capabilities in recording neural responses *in vivo* and *in vitro*. Finally, we show the versatility of the N-UNCD electrode by demonstrating stimulation on the bench-top as well as flexible manufacturing of electrode placements.

### Electrode characterization

To test for reliable fabrication of the stimulating and recording electrodes, the impedance spectra were characterized (*n* = 10, each electrode size, Figures 1B,C). As expected, the smaller recording electrodes (15 × 15 μm) showed a greater impedance magnitude than the stimulating electrodes (150 × 150 μm, Figure 1B). At a test frequency of 1 kHz, the recording electrode impedances (54.99 ± 14.7 kΩ, mean ± std) were significantly greater than for the stimulating electrodes (2.84 ± 0.63 kΩ; *p* < 0.001, Wilcoxon Rank-Sum test). In contrast, the phase of the impedances (Figure 1C) was similar between electrode types with the greatest difference occurring at lower frequencies.

### Evoked potentials recorded from the visual cortex

**Figure 2:**
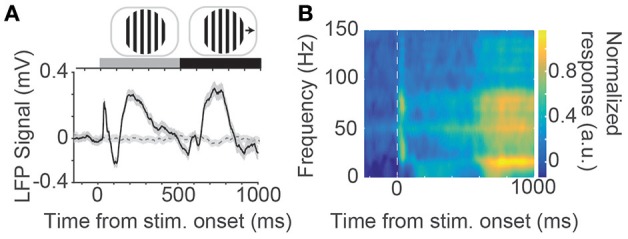
**(A)** Population average evoked response to visual grating stimuli (solid black line) measured via an N-UNCD recording electrode placed on the visual cortex. An average response across all 8 directions and trials is shown. Also shown is the average response to the control stimuli where a blank screen was displayed (dashed gray line). At the top of the figure is an example visual stimulus drifting grating that was displayed stationary for 500 ms before made to drift. **(B)** Time-frequency spectrogram of the population neural response. A large peak in the response can be seen at the onset of the stimulation and a delayed response occurs after ~700 ms (most notably the yellow patches in the 10–20 Hz band).

Next, we examined the ability of the N-UNCD electrodes (15 × 15 μm) to record neural signals from cortical tissue using an *in vivo* preparation by recording visually evoked responses from the visual cortex of a cat. Figure 2A (solid line) shows the population average response (across two animals) recorded during visual grating stimulation (Figure 2A inset). A sharp peak in the response occurs immediately after the initial stationary presentation of the gratings (~40 ms after stimulation). This response was found to be significantly different to the activity measured in the baseline period before the stimulation (320 ± 42 μV, mean ± s.e.m.; *p* < 0.001, Wilcoxon Rank-Sum test). A slower secondary peak, after approximately 200 ms, was also significantly different from baseline (389 ± 51 μV, *p* < 0.001). The SNR of this time period was 1.45 ± 0.04 (mean ± s.e.m.). When the gratings were made to drift after 500 ms, another significant peak in the recorded response was visible at approximately 200 ms after the stimulus movement (415 ± 67 μV, *p* = 0.017). In contrast, when the screen was left blank during control trials (dashed gray line) no peaks were observed.

We examined the frequency content of the recorded neural signals by calculating a time-frequency spectrogram (Figure 2B). A large response peak in the spectrogram in the gamma band (30 - 80 Hz) can be seen at the onset of the stimulation with a sustained delayed response occurring ~200 ms after stimulus movement. This increased delay is due to the sliding window of time in which the spectrum was estimated. This corresponds to the start of the movement of the sinusoidal gratings. This delayed response also showed a peak in the beta band (16 Hz) and was significantly different to the power in the baseline period (*p* < 0.001, Wilcoxon Ranksum test).

### Action potentials recorded from the retina

We tested the ability of the N-UNCD electrodes to record neural activity *in vitro*. We found that the N-UNCD recording electrodes were very sensitive to retinal activity, and spikes with high SNR could be measured. The RMS noise from the recording electrodes was approximately 2 μV in the extracellular solution.

**Figure 3:**
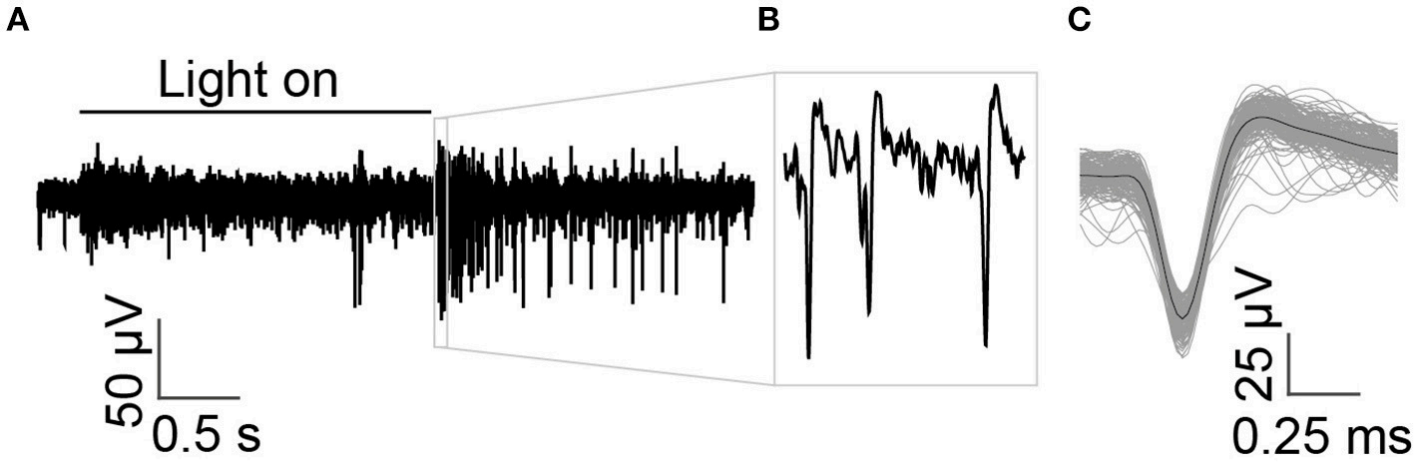
Multi-unit recordings from *in vitro* retinal ganglion cells in response to a light stimulus. **(A)** The time of the full field light stimulus is indicated with the solid black line above the recording. A cell with large spikes responds strongly at light-off and exhibits an ongoing dark response, indicating that it is an off-sustained cell. **(B)** Spikes from a single prominent sorted off-cell are shown at higher magnification. **(C)** Average shape of spikes that crossed threshold.

As the electrode was lowered onto the retina, multi-unit spikes were detected. Figure 3A shows an example recording, with spikes from multiple retinal ganglion cells. High amplitude spikes from a single cell could be easily differentiated from other cells and background noise (shown in Figures 3B,**C**). The mean spike amplitude was approximately 83 μV with SNR of 7.25.

**Figure 4:**
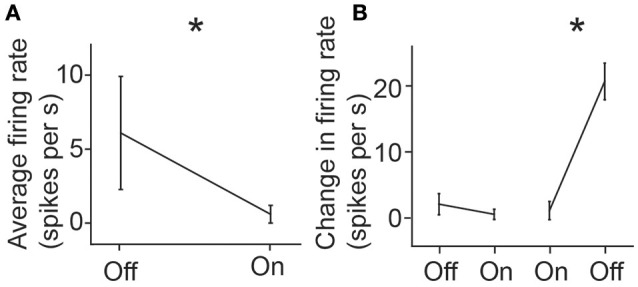
**(A)** Mean response firing rate of a retinal ganglion cell during light-on and light-off and **(B)** mean instantaneous change in firing rates during transition from light-off to on, and from light-on to off. Error bars represent standard deviations. ^*^indicates *p* < 0.05 (Rank-Sum test).

Once single-neuron activity was isolated using the N-UNCD electrodes, we characterized the retinal response to light stimulation. To do this, the mean firing rate of the cell was measured during full-field retinal periods of light-on and light-off. A significantly higher spike rate was observed during periods of light-off (OFF: 6.1 sp/s, ON: 0.6 sp/s, *p* = 0.0012, Wilcoxon Rank-Sum test; Figure 4A). In addition to this, we characterized the transient responses of the cell by measuring the changes in spike rates when the light was either turned on or off. Significant increases in the firing rates were also observed during transitions from light-on to light-off (OFF to ON: 2.1 to 0.4 sp/s, ON to OFF 1.1 to 21.0 sp/s, *p* < 0.001, Figure 4B), suggesting this cell was an off-retinal ganglion cell. These results and the *in vivo* measurements, indicate that the N-UNCD electrodes can successfully record from different types of neural tissue.

### Stimulation voltage profiles in saline

**Figure 5:**
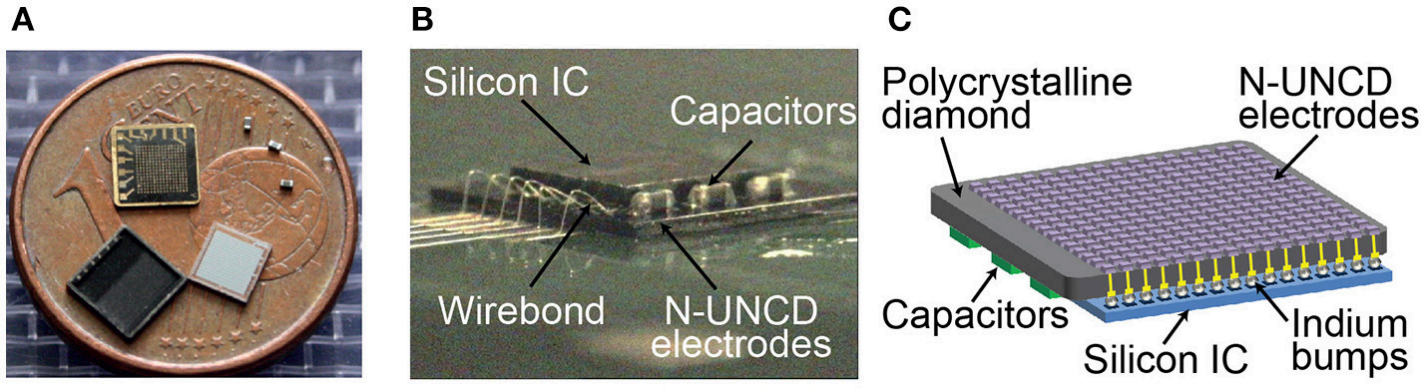
**(A)** Components of the stimulating electrode array. Diamond electrode array (shown here with metallic interconnects facing upwards), three electrical power conditioning capacitors, an ASIC chip (Tran et al., 2014), and a lid to form a hermetically sealed capsule. A 1 Euro cent coin, with a diameter of 16.25 mm, is placed for size comparison. **(B,C)** The same components integrated into a device prior to the placement of the lid. Flip chip bonding was used to connect 256 electrodes and external power/data interconnects between the ASIC and diamond array.

**Figure 6:**
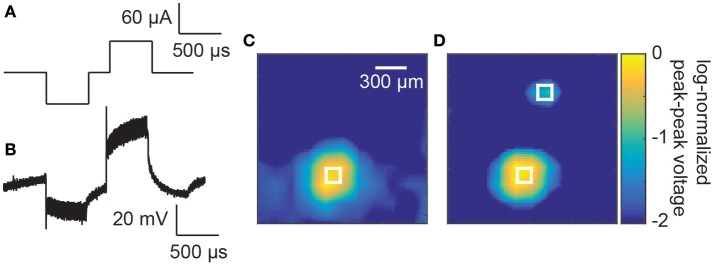
**(A)** Example stimulation waveform. **(B)** A measured voltage waveform in saline during a single biphasic current stimulus of 60 μA and phase durations of 500 μs. **(C)** Spatially smoothed and log-normalizes peak-to-peak measured voltages above the electrode array. A focussed increase in peak-to-peak voltages can be seen above the stimulating electrode in the bottom left of the image. The white square indicates the size and location of the stimulating electrode. **(D)** Measured voltages above the electrode array with the two electrodes active simultaneously (60 and 20 μA). The two white squares indicate the size and location of the stimulating electrodes (150 × 150 μm).

Finally, an array bonded to an ASIC stimulator (16 × 16 electrode array, Figure 5) was used to deliver biphasic electrical stimuli through saline (Figure 6A). The voltage profile in the saline above a stimulating electrode is shown in Figure 6B during a single stimulation event. A clear biphasic change in the saline voltage corresponding to the current injection is visible.

To test the localization of the current injection, the peak-to-peak voltages of these biphasic waveforms were measured at different locations above the stimulating electrode array. A smoothed image plot (to remove variations due to noise) of the voltage changes is presented in Figure 6C for a single stimulating electrode. A prominent spot of activity is seen in the lower left hand corner of the image corresponding to the stimulating electrode (represented by a white square of the same dimension as the electrode, 60 μA and 500 μs phases). When a second stimulating electrode was activated simultaneously with 20 μA and 500 μs phases (Hadjinicolaou et al., 2012), a secondary localized increase in voltage was measured in the top of the array (Figure 6D).

## Discussion

The aim of this work was to demonstrate the feasibility of a novel electrode material to record high-fidelity neural signals. We showed for the first time that N-UNCD is a suitable material to record neural signals with high signal-to-noise ratio in both *in vitro* and *in vivo* settings. We also demonstrated the ability to provide localized electrical stimuli through the N-UNCD electrodes. This ability to both provide stimulation and record neural signals opens possibilities for new neural prostheses based around N-UNCD electrodes.

The recorded neural responses in the *in vivo* preparations (Figure 2A) were of similar magnitude to those achieved with currently available penetrating multi-electrode arrays (100–400 μV: Wong et al., 2016), as well as those achieved by surface arrays (100–200 μV: Rubehn et al., 2009). In addition to this, we found that high SNR recordings are achievable in the *in vitro* preparations (7.25, Figure 3). While penetrating multi-electrodes will have clear advantages in spatial resolution (and the ability to record from single neurons *in vivo*), these results nonetheless indicate that N-UNCD is suitable for recording high-fidelity spiking and LFP signals, *in vitro* and *in vivo*.

These results are most likely attributed to the electrochemical impedances of the N-UNCD electrodes. A low electrochemical impedance (compared to the analog front-end impedance) is required from the recording electrodes in order to achieve a high signal-to-noise ratio (Spira and Hai, 2013; Rocha et al., 2016). As such materials with a low characteristic electrochemical impedances are required, especially since small electrode geometries needed for extracellular recordings of single or small populations of neurons result in increased impedances. In this work, the electrochemical impedances of 225 μm^2^ electrodes were below 100 kΩ in the frequency range of 100 Hz to 10 kHz (~55 kΩ at 1 kHz). This translates to a specific electrochemical impedance of ~0.11 Ω/cm^2^ at 1 kHz which compares extremely favorably to standard gold [10 Ω/cm^2^, (Kim et al., 2007)], tungsten [0.8~2.9 Ω/cm^2^, (Sankar et al., 2014)], platinum [4.9 Ω/cm^2^, (Gabriel et al., 2013)], and platinum black [0.8 Ω/cm^2^, (Gabriel et al., 2013)] electrodes. Earlier works have shown a correlation between low electrochemical impedances and high signal-to-noise ratio (Obien et al., 2015).

### N-UNCD multi-electrode arrays for neural recordings

In the last two decades, there has been a large push to record from larger areas of cortex, as well as to increase the recording density in a given area (Nicolelis et al., 2003; Kipke et al., 2008; Viventi et al., 2011; Dotson et al., 2015). Based on the present findings, electrodes fabricated from N-UNCD may be used to develop high density arrays. There is evidence that N-UNCD is well tolerated by the body over long time scales with reports of minimal histopathological reactions 15 weeks after implantation (Lichter et al., 2015). Electrodes can be fabricated to be very close to each other without the need for space to route connecting wires out of the array by integrating the feedthroughs into the design of the electrodes and integrating electrodes with CMOS electronics that can amplify and digitize the measured neural signals, as demonstrated by Ganesan et al. (2014) and Ahnood et al. (2015) for stimulating arrays. The benefit of N-UNCD is that it allows for full encapsulation with a recording chip that no other material currently offers.

However, the simplest electrode arrays manufactured from N-UNCD will be planar in shape and simply increasing the size would not result in an array suitable for implantation over large areas of the brain owing to the curvature of cortex. While N-UNCD electrode arrays could be fabricated to follow contours, the maximum contour is limited by the manufacturing process. Conformal electrode arrays, such as those fabricated using thin layers of poly(dimethylsiloxane) and dissolvable silk fibroin (Kim et al., 2010) will provide the best contact between electrodes and the brain over a large area of cortex. To overcome this limitation, a similar approach to that used with current penetrating microelectrode arrays, where multiple individual arrays are “tiled” across cortical areas, may mitigate the problem of flat, rigid arrays.

While we have successfully demonstrated recording of neural signals from the surface of the cortex, modification of the electrode pillars to protrude further from the base of the array is feasible. This may eventually allow for the electrodes to penetrate the cortex to record action potentials from neurons in deeper cortical layers. Nevertheless, significant work is required to increase the longevity of the electrodes and minimize tissue damage from electrode insertion (Polikov et al., 2005; Barrese et al., 2016).

### High channel-count, closed-loop prostheses

In this work, we demonstrated the recording capability of N-UNCD, and flexibility of this material for creating high density arrays (Figure 5). We propose that the ability to flexibly design electrodes of different surface areas and dimensions will allow for the development of N-UNCD-based, closed-loop prosthetic devices. Closed-loop devices that can tailor electrical stimulation based on the inferred state of the underlying tissue from recorded biological signals, such as the local field potential (LFP), may greatly increase the efficacy of neural prostheses (Kameneva et al., 2014). Such devices could have significant impact on the detection and suppression of epileptic seizures (Berényi et al., 2012; Ramgopal et al., 2014), provide more punctate phosphenes for cortical-based or retinal vision prostheses (Torab et al., 2011; Wong et al., 2016; Halupka et al., 2017), provide sensory feedback for brain-machine interfaces (O'Doherty et al., 2011), and provide more efficacious functional electrical stimulation (Loeb et al., 1991). Moreover, diamond surfaces provide a rich surface chemistry platform for sensing of neurochemicals (Chan et al., 2009; Bennet et al., 2016), which can extend the capability of a sensing electrode beyond the simple detection of electrical activities of neurons.

While biocompatibility, encapsulation, recording, and stimulating capabilities are beneficial, the stiffness of the material may be a significant hurdle in clinical translation (Subbaroyan et al., 2005). There has been a recent push for the development of more flexible electrode technologies (Kozai et al., 2012; Apollo et al., 2015). As such, the use of N-UNCD arrays may be limited to acute implantations at this stage. However, it may be possible to embed N-UNCD electrodes in a flexible poly(dimethylsiloxane) substrate to increase the conformability (Bergonzo et al., 2011).

Another major issue, for closed-loop devices is dealing with recording artifacts that arise when electrical stimulation is delivered. However, there are many artifact removal techniques that could be implemented in hardware (O'Keeffe et al., 2001; Heffer and Fallon, 2008) as well as established methods to interleave stimulation and recording intervals with achievable rates of up to 20 Hz (O'Doherty et al., 2011). Finally, even though N-UNCD electrodes have a high charge injection capacity, the minimum size of electrodes that can be fabricated for chronic *in-vivo* use will ultimately be limited by the maximum charge density allowable in the target tissue before injury occurs (McCreery et al., 1990).

In the current work, no recordings or stimulation of neural tissue was performed with the 16 × 16 electrode array and ASIC due to difficulties in implantation and interfacing with neural tissue. In order to make this device feasible for in high-channel count *in vivo* work, further development is required into methods to implant and stabilize the device on the cortical surface. Regardless of these issues, the recording and stimulating capabilities still offer significant advantage especially in the acute experimental domain.

### *In vitro* based multi-electrode arrays

Another application for N-UNCD electrodes is *in vitro* microelectrode arrays (Oka et al., 1999; Spira and Hai, 2013). While no recordings were performed with multi-electrode arrays in this study, the single N-UNCD electrode *in vivo* recordings show neural activity can be recorded with a high signal-to-noise ratio (Figure 3). These recorded responses were similar in amplitude to those reported previously using other technologies [~100 μV: (Grumet et al., 2000)], as well as with similar latencies (Fries et al., 2001). For this application, the mechanical flexibility of the material is not an issue and the benefit of integration with CMOS circuitry becomes more important (Hierlemann et al., 2011).

A further benefit of this material for constructing multi-electrode arrays is that the insulating polycrystalline diamond substrate can be transparent over a wide optical window with wavelength range of 225 nm to 10 μm and above (Mildren, 2013). This would allow for light to be transmitted through the array, improving the ease of use with microscopes. The insulating diamond has a wide optical window compared to glass allowing for the use of wavelengths from ultraviolet to deep infrared.

## Conclusion

In this work, we demonstrated that N-UNCD electrodes can record high signal-to-noise ratio signals from neural tissue and can deliver focused electrical stimulation. N-UNCD electrodes were shown to be highly flexible in their application with successful recordings of action potentials from single neurons in an *in vitro* retinal preparation, as well as local field potential responses from *in vivo* visual cortex. Moreover, earlier works have demonstrated the efficacy the same N-UNCD electrodes for stimulation of neuronal cells. As shown in this work, the stimulating and recording electrodes can be fabricated side by side as a single array. As well as being bio-stable and bio-compatible, N-UNCD electrodes offer both a high specific charge injection capacity and low specific electrochemical impedance, both prerequisites for high quality stimulating and recording electrodes. Moreover, as demonstrated here, the array can be integrated with silicon-based integrated circuits using vertical interconnects, which enable high density arrays. The ease and scalability of fabrication of diamond electrodes with custom sizes, as well as direct bonding of silicon integrated circuits to the electrodes, allows for versatile neural prostheses that can both record and stimulate. New devices fabricated from these materials could allow for more efficacious closed-loop prostheses.

## Author contributions

YW, AA, MM, HM, SP, MI, DG, and AB designed the studies. YW, AA, WK, KG, HM, and SP contributed to the design and manufacturing of the devices. YW, AA, MM, WK, HM, and MI performed the experiments. YW, AA, MM, MI, DG, and AB helped analyze and interpret the data. YW and AA wrote the manuscript with input from all authors. All authors discussed the results.

### Conflict of interest statement

SP is a shareholder, director and chief technology officer of iBIONICS, a company developing a diamond based retinal implant. MM and SP are directors and shareholders in Carbon Cybernetics, a company developing a carbon based brain machine interface. The remaining authors declare that the research was conducted in the absence of any commercial or financial relationships that could be construed as a potential conflict of interest.
